# Development of a Physical Activity Maintenance intervention for people with PERsistent musculoskeletal pain (PAMPER): a mixed-methods study protocol

**DOI:** 10.1136/bmjopen-2025-103763

**Published:** 2025-06-10

**Authors:** Gregory Booth, Lindsay Bearne, Danielle D’Lima, Mohammed Hudda, Michael Ussher

**Affiliations:** 1 Therapies Department, Royal National Orthopaedic Hospital NHS Trust, London, UK; 2 Population Health Research Institute, School of Health and Medical Sciences, City St George’s University of London, London, UK; 3 Centre for Behaviour Change, Department of Clinical, Educational and Health Psychology, University College London, London, UK; 4 Department of Population Health, Dasman Diabetes Institute, Kuwait City, Kuwait; 5 Institute of Social Marketing and Health, University of Stirling, Stirling, UK

**Keywords:** Chronic Pain, Musculoskeletal disorders, Exercise, PAIN MANAGEMENT

## Abstract

**Abstract:**

**Introduction:**

Persistent musculoskeletal pain is a leading cause of disability and need for rehabilitation globally. Many people with the condition attend pain management programmes (PMPs) for rehabilitation and support with self-management. Physical activity (PA) is an essential self-management strategy facilitated on PMPs as it benefits symptoms, general health and well-being. PA needs to be maintained in the long term to continue to be beneficial. However, while many patients increase their PA during or immediately after a PMP, they commonly find it difficult to maintain it in the long term. This study aims to address this problem by developing an intervention to support PA maintenance after a PMP.

**Methods and analysis:**

This mixed-methods study will be guided by the Medical Research Council guidelines for developing complex interventions and the Behaviour Change Wheel intervention development framework. Participants will be recruited from multiple UK National Health Service PMPs. Participants will include patients with persistent musculoskeletal pain who have completed PMPs, their PA partners (people who support them with PA) and healthcare professionals who facilitate PA on PMPs. The study will be conducted in three phases. In phase 1, qualitative interviews will explore the experiences, barriers and facilitators of PA maintenance after a PMP and potential characteristics for a PA maintenance intervention from patient, PA partner and healthcare professional perspectives. Phase 2 will consist of a prospective longitudinal pilot study to identify factors associated with PA maintenance after a PMP. Phase 3 will involve developing a logic model and co-designing the intervention with patient, PA partner and healthcare professional stakeholder groups.

**Ethics and dissemination:**

The project received research ethics committee (REC) and Health Research Authority approval on 4 June 2024 (REC: North West—Liverpool Central, REC reference: 24/NW/0174, IRAS Project ID: 340674). Findings will be disseminated by peer-reviewed publications, conference presentations, social media and lay summaries for patients and the public.

**STRENGTHS AND LIMITATIONS OF THIS STUDY**

- This study has been informed by patient and public involvement (PPI) and it has an established PPI group that will be key contributors throughout.
- The study is multicentre, multiphase and mixed methods, increasing its rigour and generalisability.
- Multiple stakeholders, including patients and healthcare professionals, will be included throughout the intervention development.
- Phase 2 is a pilot study with an anticipated relatively small sample size and short follow-up.

## Introduction

 Physical activity (PA) has substantial benefits for people with persistent musculoskeletal pain; it improves their pain, physical function, mental health, quality of life and general health, and reduces the impact of common comorbidities.^1^,^8^ PA has been defined as any movement produced by skeletal muscles that requires energy expenditure, for leisure, transport, work or exercise.^9 10^ Persistent musculoskeletal pain is a disabling condition characterised by pain lasting 3 months or longer in bones, joints, muscles, tendons and other soft tissues.^11^ Musculoskeletal conditions are a leading cause of disability^12^ and are the most common reason for people needing rehabilitation globally.^13^ Musculoskeletal conditions cost the UK National Health Service (NHS) approximately £5 billion annually,^14^ and 38% of people with a musculoskeletal condition are economically inactive.^15^ People with the condition often have low PA levels.^14^

Pain management programmes (PMPs) are multidisciplinary interventions that provide rehabilitation and teach self-management strategies to patients with persistent pain.^16^ Implementing self-management requires initial behaviour change and then behaviour maintenance to continue the benefits.^17 18^ PA is a core self-management strategy promoted on PMPs.^16^ However, while PMPs improve day-to-day function and quality of life, these effects can be short lived, as maintenance of self-management strategies, including PA, remains challenging for people with persistent musculoskeletal pain.^19^ Furthermore, PA interventions for people with persistent musculoskeletal pain provide short-term effects on PA levels, but PA is not maintained beyond immediate post-intervention.^20^

There is currently no agreed conceptual or operational definition of PA maintenance.^21^ One approach defines PA maintenance by achieving universal thresholds such as the WHO’s PA guidelines.^10 21^ This approach does not consider individual differences in PA levels and disability and may not be acceptable to people with musculoskeletal conditions.^22^ Another approach defines PA maintenance as the achievement of a personal PA goal.^23^ This better respects individual differences in PA levels and individuals’ goals towards PA, which are common among people with persistent musculoskeletal pain, and will be used throughout this study.^23^

The barriers and facilitators to PA uptake and maintenance are likely to be different, and therefore, different interventions are required for each stage.^17^ PA maintenance interventions are ‘secondary interventions specifically aimed at ongoing participation following an initial uptake intervention’.^24^ They have shown promise for people with cardiac conditions following cardiac rehabilitation, but to our knowledge, none have been developed or tested in people with persistent musculoskeletal pain.^24^ Current evidence of barriers and facilitators to PA for people with persistent musculoskeletal pain has focused on PA in general and not specifically uptake or maintenance, and it is unclear which barriers and facilitators are more important for uptake versus maintenance of PA.^25^,^27^ Knowledge of barriers and facilitators is essential for designing effective interventions.^28^ Similarly, several behaviour change techniques (BCTs) support PA uptake, but whether the same BCTs are useful for PA maintenance is unclear.^2029^,^31^

An intervention aimed at improving PA maintenance has the potential to improve self-management and health outcomes for this population.

### Aims and objectives

The overall aim of this study is to develop an intervention to support people with persistent musculoskeletal pain to maintain PA, after completing a PMP (a PA uptake intervention), to enhance long-term self-management.

The objectives of this mixed-methods study are to:

1. Qualitatively explore the experiences of and barriers and facilitators to PA maintenance after PMPs for people with persistent musculoskeletal pain.
2. Qualitatively explore potential characteristics (eg, BCTs, delivery methods) of an intervention to support PA maintenance after PMPs for people with persistent musculoskeletal pain.
3. Quantitatively identify factors associated with PA maintenance over 6 months after a PMP for people with persistent musculoskeletal pain.
4. Co-design a theoretically informed intervention to support PA maintenance after a PMP with a patient and clinical codesign group.

## Methods and analysis

### Study design

**Figure 1:**
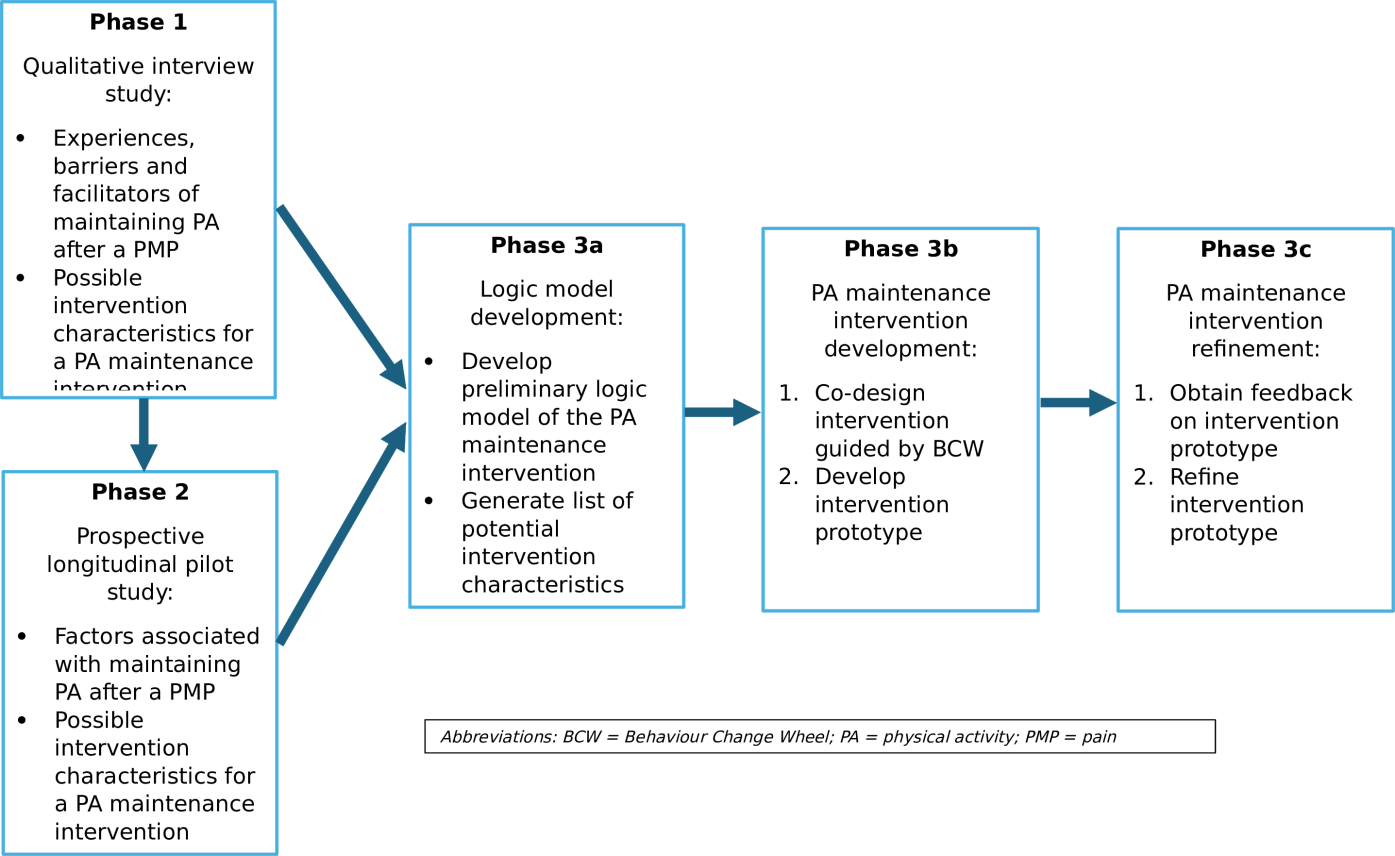
Physical Activity Maintenance intervention for people with PERsistent musculoskeletal pain project diagram.

This study will use a mixed-methods exploratory sequential approach,^32^ consisting of qualitative, quantitative and participatory methodology, across three phases. Triangulating data from multiple sources and using a variety of methods will strengthen our understanding of behaviour maintenance.^28^ Therefore, phase 1 will use qualitative methods and phase 2 will use quantitative methods (informed by phase 1) to comprehensively explore the factors that influence PA maintenance. Participatory methods will be used to codesign the intervention prototype in phase 3 (figure 1).

This study will be situated within the pragmatist research paradigm as it is focused on the practical implications of the research by using mixed methods to design an intervention aimed at improving healthcare and patient outcomes.

The full study protocol has been registered on the Open Science Framework (https://osf.io/jq95b).

### Theoretical framework

This research will be underpinned by several frameworks. The Medical Research Council guidelines for developing complex interventions recommend considering the context, developing and refining theory, engaging stakeholders, identifying key uncertainties and refining interventions.^33^ The Behaviour Change Wheel (BCW)^34^ is an evidence-based, theoretical, systematic framework for developing behavioural interventions and has been used in the development of PA interventions for multiple populations.^35^,^37^ At the hub of the BCW is the Capability, Opportunity, Motivation-Behaviour (COM-B) model, which explains how capability, opportunity and motivation interact to generate behaviour. COM-B is integral to intervention design as it supports identification of what needs to be targeted in the intervention. The Theoretical Domains Framework (TDF), which maps to COM-B, defines 14 domains for categorising barriers and facilitators and gives a more detailed understanding of behaviour.^38^ COM-B can explain how TDF domains interact with each other, which enables generation of theory.^28 38^ In the following BCW steps, intervention types (eg, enablement), policy types (eg, service provision), BCTs (eg, action planning) and modes of delivery (eg, a website) are selected.^34^

### Setting

This study will recruit participants from the following NHS PMPs in England.

1. Royal National Orthopaedic Hospital NHS Trust.
2. Guy’s and St Thomas’ NHS Foundation Trust.
3. The Walton Centre NHS Foundation Trust.
4. Oxford University Hospitals NHS Foundation Trust.

### Phase 1: identifying barriers and facilitators to PA maintenance after a PMP and potential intervention characteristics

#### Study design

This qualitative interview study will explore the experiences, barriers and facilitators to PA maintenance and identify possible intervention characteristics (eg, BCTs) for supporting PA maintenance for people with persistent musculoskeletal pain after completing a PMP.

#### Participants

##### Inclusion criteria

1. Patient participants: adults (≥18 years of age) with persistent musculoskeletal pain (pain≥3 months in bones, joints, muscles, tendons or other soft tissues)^11^ who have completed a PMP (a programme adhering to the British Pain Society’s PMP guidelines)^16^ 6–18 months prior to study enrolment.
2. PA partners of patient participants: any adult who supports the patient with their PA or someone they do PA with.
3. Healthcare professionals working on PMPs: any healthcare professional involved in facilitating PA during a PMP (eg, physiotherapists, occupational therapists and psychologists).

##### Exclusion criteria

1. People with persistent musculoskeletal pain who also have a health condition where moderate-to-vigorous PA is contraindicated (eg, unstable/uncontrolled cardiac conditions).
2. People with persistent musculoskeletal pain who are pregnant (as pregnancy affects PA and they would require a separate intervention).
3. People with persistent musculoskeletal pain and PA partners who are unable or unwilling to provide informed consent.

### Sampling strategy and sample size

Purposive sampling will be used for patient participants and healthcare professionals. For patient participants, this will ensure that participants represent people who are both regularly physically active (n=12) and inconsistently active/inactive (measured through self-identification) (n=12). We will also aim for maximum variation in other participant characteristics (including diagnoses, pain sites, duration of pain, age, gender, ethnicity, educational qualifications, employment status, PMP completed, time since PMP and whether they have a PA partner or not). Recruitment of PA partners will be based on nomination by patient participants.

For healthcare professionals, purposive sampling will be used to ensure participants’ represent different PMPs, professions, grades, time working on PMPs and genders. We anticipate requiring up to 12 healthcare professionals.

Participants will be screened using the purposive sampling criteria prior to obtaining written informed consent. The sample size will be guided by the principles of information power and continuously evaluated as data generation and analysis progress.^39^

### Recruitment

Patient participants will be invited to participate either by being given a participant information sheet (PIS) by a clinician when attending PMP follow-up appointments or by being sent the PIS by a clinician by email or post. Potential participants will be invited to email the research team if they are interested in participating. The researchers will then discuss the study with the patient and screen their eligibility over the telephone, including eligibility towards satisfying the sampling strategy. If selected for interview, the researchers will contact the patient by telephone to give them an opportunity to ask further questions and invite them to attend the interview with a PA partner (ie, a person they nominate who has supported them with their PA). They will then be sent (by email or post, depending on patient preference) a consent form for themselves and, if applicable, a PA partner PIS and consent form. After signing and returning the consent form(s) (by email or post), the patient, and if applicable, their PA partner, will be asked additional questions, by the research team, over the telephone relating to their personal characteristics (marital status, whether they live alone or with others, and comorbidities for patient participants, and age, gender, ethnicity, employment status, educational qualifications and health conditions for PA partners) and an interview will be arranged at a time that is convenient for the participants.

Healthcare professionals working on PMPs will be invited to participate by the principal investigators at their site by an email containing the PIS. Healthcare professionals will be invited to contact the lead researcher by email. The researcher will then contact them by email or by telephone to answer questions about the study and to assess their eligibility. If they are selected for interview, they will be emailed a consent form. Once written informed consent has been obtained, an interview will be arranged.

### Data generation

Patient participants without PA partners, and healthcare professionals, will participate in individual interviews. Patient participants with PA partners will participate in dyadic interviews.

Interviews, lasting approximately 45 min, will be conducted by GB (a senior pain management physiotherapist and doctoral candidate). They will be in-person (on participating healthcare provider or university premises), on the telephone or via video call, depending on participant preference. They will be audio recorded and transcribed verbatim using Otter.ai software (http://www.otter.ai). To facilitate research inclusion, interpreters will be available when required. A professional transcription company will transcribe any interviews where an interpreter is used.

Topic guides for patients/PA partners and healthcare professionals have been developed and piloted collaboratively with the research team and patient and public involvement (PPI) members. To ensure comprehensive exploration of barriers and facilitators to PA maintenance, the topic guides include questions relating to the 14 TDF domains.^38^ Broader, open questions are included to explore perspectives that relate to non-TDF domains.^40^ Questions relating to intervention characteristics have been informed by the Template for Intervention Description and Replication (TIDieR) checklist to ensure all key intervention characteristics are considered.^41^ Topic guide topics are summarised in table 1 and an iteration of the topic guides is displayed in online supplemental material file 1.

**Table 1:** Phase 1 interview topic guide topics

Patient and PA partner topic guide	Healthcare professional topic guide
Current PA (activities, location and level)	Perceptions of how well patients maintain PA after a PMP
Experiences of maintaining PA since PMP	Patients’ feelings about maintaining PA long term
Feelings about maintaining PA long term	Barriers and facilitators to patients maintaining PA long term, after a PMP
Barriers and facilitators to maintaining PA since PMP	
Sections relating to TDF domains: Planning, intentions, routine, competing demands, burden of PA (TDF domains: intentions; goals; optimism; reinforcement; behavioural regulation; memory, attention and decision processes; environmental context and resources; social/professional role and identity) Confidence related to long-term PA (TDF domains: skills, beliefs about capabilities, optimism) Feelings when doing PA (TDF domain: emotions) Problem-solving, memory regarding PA and PA monitoring (TDF domains: skills; memory, attention and decision processes; behavioural regulation) Knowledge and beliefs about maintaining PA (TDF domains: knowledge, beliefs about consequences) Social influences and communication (TDF domains: skills, social influences)	Sections relating to TDF domains: Planning, intentions and goals towards PA (TDF domains: intentions, goals, reinforcement, behavioural regulation) Confidence related to long-term PA (TDF domains: beliefs about capabilities, optimism) Problem-solving, memory regarding PA and PA monitoring (TDF domains: skills; behavioural regulation; memory, attention and decision processes) Knowledge and beliefs about maintaining PA (TDF domains: knowledge, beliefs about consequences) Social influences and communication about PA (TDF domains: social influences, skills) Other support—healthcare professionals and community-based PA (TDF domain: environmental context and resources)
Intervention characteristics What the intervention could include Who could provide the intervention How the intervention could work Where the intervention could be used When the intervention is needed How much of the intervention is needed Personalisation/tailoring the intervention	

PA, physical activity; PMP, pain management programme; TDF, Theoretical Domains Framework.

### Data analysis

Data generation and analysis will be conducted concurrently. Data analysis will be supported by QSR NVivo software.^42^ Data relating to barriers and facilitators will be analysed first using inductive reflexive thematic analysis.^43^ The themes and subthemes will then be mapped onto the TDF,^38^ and then TDF domains will be mapped onto COM-B.^28^ Preliminary descriptive analysis of the first 10 interviews will be used to identify variables that will be assessed in phase 2.

The intervention characteristics will initially be analysed using inductive reflexive thematic analysis,^43^ and the themes and subthemes will then be mapped onto the TIDieR checklist,^41^ BCW^34^ and BCT ontology.^44^

Respecting researcher subjectivity as the primary tool in reflexive thematic analysis, GB will reflect on their positionality and experience and the way this may influence data generation and analysis throughout the study.

### Phase output

The barriers and facilitators identified in this phase will inform the variables assessed in phase 2 and the intervention targets in phase 3. The identified intervention characteristics will support intervention development in phase 3.

### Phase 2: factors associated with PA maintenance after a PMP and potential intervention characteristics

#### Study design

This prospective longitudinal pilot study will identify factors associated with PA maintenance over 6 months post-PMP and identify potential intervention characteristics.

#### Participants

##### Inclusion criteria

1. Adults (≥18 years of age) with persistent musculoskeletal pain (pain≥3 months in bones, joints, muscles, tendons or other soft tissue^11^) who have completed a PMP.

##### Exclusion criteria

1. People who have a comorbid health condition where moderate-to-vigorous PA is contraindicated (eg, unstable/uncontrolled cardiac conditions).
2. People who are pregnant.
3. People who are unable or unwilling to provide informed consent.

### Sampling strategy and sample size

Convenience sampling will be used to maximise recruitment. We aim to recruit 150 participants. This is a pragmatic consideration based on what is estimated to be feasible within the planned 6-month recruitment period with four sites participating, and that will provide sufficient data for the study.

The primary analysis will investigate the association between baseline scores and PA maintenance (achievement of a personal PA goal^23^). Power calculations were conducted to assess whether the sample size target of 150 participants would be sufficient to detect meaningful associations in a logistic regression model for investigating this primary analysis. Assuming a significance level of 0.05, a power of 0.80, a 60:40 ratio of individuals who maintain PA to those who do not^45 46^ and an expected OR of 1.60, the target sample size of 150 was deemed adequate to detect meaningful associations. This estimated OR of 1.60 is plausible when reviewing the literature assessing associations between self-efficacy (a common factor associated with PA levels) and PA maintenance.^45 47^

### Recruitment

Recruitment is planned for 6 months. Patients will be invited to participate and given a PIS by the site clinical teams towards the end of their PMPs. After receiving the PIS, patients will be given at least 24 hours to consider participation. There will be multiple possible methods for obtaining informed consent:

1. Site clinical teams screen the patient and obtain informed consent from the patient before they complete their PMP and forward the patients’ contact details and screening information to the research team.
2. Patients complete a consent to contact form online (accessed via a QR code) or on paper, depending on participant preference. The research team will then call the patient after they have finished their PMP to discuss the study further, screen for eligibility and seek consent either electronically or by post, depending on their preference.
3. The patient expresses interest by emailing the lead researcher directly using an email address provided on the study invitation letter and PIS. The research team will then call the patient after they have finished their PMP to discuss the study further, screen for eligibility and seek consent either electronically or by post, depending on their preference.

### Measures and outcomes

#### Primary outcome

All measures will be completed at baseline and 6-month follow-up. The primary outcome is the achievement of a personal PA maintenance goal (binary outcome). PA will be measured for four consecutive weeks at both timepoints. After the baseline assessment, participants will review how much PA they did each week with the research team and set a weekly PA goal, in minutes per week of PA, that they aim to achieve for the study duration. This goal cannot be lower than the lowest amount of PA measured in one week during the baseline measurement period. At the 6-month follow-up, to be classed as having maintained their PA, participants must have achieved their goal for at least three of the four PA measurement weeks.

Objective PA will be measured using a triaxial GENEActiv wrist-worn accelerometer, which is a reliable and valid device for measuring PA in adults.^48^ The GENEActiv is small and waterproof, and participants are blinded to data collection, which will reduce the chance of the accelerometer influencing PA behaviour. The GENEActiv will sample continuously at a frequency of 20 Hz.

#### Independent variables

The assessed independent variables (sociodemographic and clinical characteristics and common barriers and facilitators identified in phase 1) will be measured using bespoke questionnaires for baseline and 6-month follow-up, which were developed following preliminary analysis of the first 10 phase 1 interviews. There were multiple stages to developing the questionnaires: first, descriptive analysis of the first 10 phase 1 interviews was conducted by creating groups of similar codes to identify barriers and facilitators. Our previous systematic review of barriers and facilitators to PA in people with persistent musculoskeletal pain was then reviewed to ensure potential barriers and facilitators were comprehensively covered.^25^ The research team then agreed on the barriers and facilitators to be included in the questionnaires. Validated questionnaires relating to the barriers and facilitators were then sought and reviewed. Where a validated questionnaire existed, only individual items relevant to the identified barriers and facilitators were included. These items were then adapted so that they asked about PA maintenance, and so the measurement scales were consistent throughout the questionnaire. When no appropriate questionnaire items existed or could be found, the research team devised questions. After a first draft of the questionnaire was developed, the PAMPER PPI group were consulted regarding the wording of questionnaire items. They were asked if the questions asked what the research team intended which were amended accordingly to enhance face validity. They were also asked to recommend changes to the wording of questions to ensure they were appropriate and understandable to participants, and they piloted the questionnaires to test the completion time. The questionnaire development was an iterative process between the research team and the PPI group and undertaken until a final version was agreed on. See onlinesupplemental material files 2  3 for the questionnaires. The topics of questions in the questionnaires are listed below. Where a questionnaire was adapted from a validated questionnaire, the source questionnaire is referenced:

1. Confidence regarding the ability to maintain PA.
2. Confidence recovering from lapses in PA level.
3. Self-efficacy for PA maintenance (eg, when pain is higher).^49^
4. Prioritising PA despite competing demands (eg, work).
5. Importance of PA maintenance to them.
6. Motivation for PA maintenance.
7. Goals for PA maintenance.
8. Perceived benefits of PA.^50^
9. Beliefs about PA (eg, relating to harm).^51^
10. Ability to remember PA.
11. How automatic PA is.^52 53^
12. Planning PA
13. Pacing PA
14. Managing pain so they can do PA.
15. Influence of pre-pain PA level.
16. Access to places and equipment.
17. Being able to afford PA financially.
18. PA instructors.
19. Social media influence.
20. Adapting PA when required
21. Monitoring PA.

Data on participants’ sociodemographic and clinical characteristics, to be collected via the baseline questionnaire, will include age (years), gender, ethnicity, pain diagnosis, pain sites, duration of pain, comorbidities, educational qualifications and which PMP they completed. Employment status, whether they have a PA partner or not, whether married/living with a partner and whether they live alone or with others, will be measured at baseline and 6-month follow-up to identify any changes. All sociodemographic and clinical data will be self-reported.

We will also collect data on recruitment (eg, numbers screened) and questionnaire and PA measurement completion rates.

#### Qualitative questions

The 6-month questionnaire will also ask four open questions. This will include two questions regarding barriers and facilitators participants experienced during the study period and two questions regarding possible intervention characteristics (what could be included and how could it be delivered) for the PA maintenance intervention.

### Data collection

After returning the consent form, participants will either be sent a link to complete the baseline questionnaire online using Microsoft Forms or posted a paper questionnaire and prepaid envelope, depending on their preference. The accelerometers will be sent and returned by post. Participants will be provided with detailed instructions on how to use the device and will be asked to wear it at all times. A similar approach will be used to collect data at the 6-month follow-up.

### Data analysis

PA will be reviewed for each participant after the 6-month follow-up and compared with the goal set following baseline assessment to assess if they achieved PA maintenance.

Descriptive statistics will be used to summarise demographic and clinical characteristics, PA (total and light, moderate and vigorous intensities) and independent variables at baseline and follow-up. For continuous variables, the distribution of variables will be reviewed and presented as means and SDs if normally distributed, or medians and IQRs if the distribution is skewed. Categorical variables will be presented as frequencies and percentages. Multilevel logistic regression will be used to identify factors associated with PA maintenance while accounting for nested study design by site and reported as ORs. This analysis will be adjusted for confounders of PA maintenance, including age and gender. Each potential barrier/facilitator will be analysed independent of each other, adjusting for potential confounders. All statistical analyses will be conducted using Stata.

Data from open questions will be analysed using the same approach as phase 1.

### Phase outputs

Factors that are associated with PA maintenance will be considered as intervention targets in phase 3, along with the findings from phase 1. Identified intervention characteristics will inform intervention development in phase 3. This phase will also enable assessment of the direction of association for each factor to inform a larger future study to definitively assess the strength of associations.

### Phase 3a: development of a preliminary logic model for a PA maintenance intervention

Findings from phases 1 and 2, including barriers, facilitators and intervention characteristics, will be triangulated to develop a preliminary logic model, guided by the BCW. Following the steps of the BCW, the logic model will include the potential barriers and facilitators to be addressed, the intervention types, BCTs and modes of delivery. The logic model will be developed and refined throughout the following phases.

### Phase 3b: co-design of an intervention to support PA maintenance in people with persistent musculoskeletal pain after a PMP

#### Study design

Guided by the BCW,^34^ participatory methods will be used to systematically co-design the PA maintenance intervention with a patient, PA partner and clinical stakeholder group.

#### Participants

Eligibility criteria for patient participants, PA partners and healthcare professionals will be as per phase 1.

#### Sampling strategy and sample size

Purposive sampling will be used for patient participants and healthcare professionals as per phase 1. The co-design group will include 8–12 people with persistent musculoskeletal pain who have completed a PMP and, where available, their PA partners and 6–8 healthcare professionals working on PMPs. These numbers will allow satisfaction of the sampling criteria, will be a manageable group size and will allow balance and equality in contributions across the groups.

Participants will be screened using the purposive sampling criteria prior to consent.

#### Recruitment

Recruitment for patient participants, PA partners and healthcare professionals will be conducted as per phase 1.

#### Data collection and analysis

Several half-day co-design workshops will be conducted. The first workshops will be separate patient (and PA partner) and healthcare professional groups to encourage each group to speak openly about their needs and priorities for the intervention. The following workshops will be joint patient, PA partner and healthcare professional groups to collaboratively design the intervention.

The workshops will either be in-person or online using videoconferencing software. They will systematically and iteratively work through the stages of the BCW framework,^34^ deciding which barriers and facilitators will be targeted, what intervention types and BCTs will be used and how and when the intervention will be delivered. This will include identifying core and optional intervention components to facilitate tailoring. The mechanisms for tailoring the intervention will be decided on during the workshops.

Examples of possible activities within the workshops include consensus exercises, surveys, rating different components or brainstorming activities. Triangulated findings from phases 1 and 2, BCW matrices linking intervention targets to intervention types and intervention types to BCTs,^28^ existing evidence on PA maintenance interventions for other conditions and BCTs, the Acceptability, Practicality, Effectiveness, Affordability, Side effects, Equity (APEASE) and the TIDieR checklist will be brought into the workshops to support decision making regarding the intervention. The language used throughout the workshops will be in lay terms.

The specific design of the workshops and activities within the workshops will be designed by the research team and the PAMPER PPI group prior to the workshops starting and will be informed by the previous phases.

An intervention prototype will be developed by the research team and PPI group members on completion of the workshops.

### Phase 3c: feedback and refinement of the intervention prototype

#### Study design

A single, half-day workshop will be conducted with a patient, PA partner and clinical stakeholder group to generate feedback and refine the intervention.

#### Participants

Inclusion and exclusion criteria for patient participants, PA partners and healthcare professionals will be as per phases 1 and 3b.

#### Sampling strategy and sample size

Participants from phase 3b will be invited to participate, as well as new participants, to gather fresh perspectives on the intervention. Purposive sampling as per phases 1 and 3b will be used to select the participants. We aim to recruit up to eight participants from phase 3b: up to four people with persistent musculoskeletal pain (and additionally, their PA partners) and up to four healthcare professionals. Up to eight new participants will be recruited: up to four people with persistent musculoskeletal pain (and additionally, their PA partners) and up to four healthcare professionals. Participants will be screened using the purposive sampling criteria prior to consent.

#### Recruitment

New patient, PA partner and healthcare professional participants will be recruited as per phases 1 and 3b.

All participants from phase 3b who consented to be contacted about phase 3c will be invited to participate by email from the research team containing the PIS. Those who have not replied within one week will be called to assess their interest and answer any questions. To volunteer, they will email the research team. They will then be provided with a consent form to sign and return, by email or post, depending on their preference.

#### Data collection and analysis

The workshop will either be in-person or via videoconferencing. Prior to the workshop, participants will be sent the intervention prototype with time to familiarise themselves with it. In the workshop, participants will discuss the whole intervention and each component, providing feedback and suggestions for improvement. Discussions will be guided by the APEASE criteria^28^ and TIDieR checklist.^41^ The logic model and intervention prototype will be refined by the research team and PPI group following the workshop.

### Patient and public involvement (PPI)

The project has a PPI group consisting of people with persistent musculoskeletal pain who have completed PMPs. This group helped to identify the overall aim of the research and supported the study design prior to the funding application in January 2023. They will continue to support the refinement of each phase as the study progresses (eg, the co-design activities). The PPI group has helped develop the participant-facing materials that will be used in recruitment (eg, PISs). They have helped develop and pilot the topic guide for phase 1 and supported the development of the questionnaires for phase 2. Throughout the project, they will assist with developing patient/public-facing dissemination materials (eg, lay summaries).

### Ethics and dissemination

The project received research ethics committee (REC) and Health Research Authority approval on 4 June 2024 (REC: North West—Liverpool Central, REC reference: 24/NW/0174, IRAS Project ID: 340674). Informed consent will be obtained from all participants prior to taking part.

This is a low-risk study. There is potential for patient participants to experience distress during the phase 1 interviews, when discussing their PA. The lead researcher is a senior physiotherapist with many years of experience working with people with persistent pain and will use their clinical experience to assess and manage participant distress, signposting them to appropriate services/support when required.

Patient and PA partner participants will be offered shopping vouchers for their participation in phases 1, 3b and 3c and on return of the accelerometers and completion of the follow-up questionnaire in phase 2. Healthcare professionals’ employers will be reimbursed for their attendance at work package 3b and 3c workshops. All participants will be reimbursed for travel costs associated with their participation.

All data will be processed according to the Data Protection Act, the General Data Protection Regulation and data protection policies at the Royal National Orthopaedic Hospital NHS Trust, which is the sponsor of this project. Data for each participant will be identified by a unique study participant number and kept separately from personally identifiable data to ensure confidentiality. Personalised data will only be accessible to the study team or other authorised personnel.

The results of each phase will be published in scientific journals and presented at national and international conferences to maximise the reach to clinical and academic audiences. Lay summaries of the results will be written for patients and other members of the public. Regular project updates will be available on the PAMPER project website (URL: www.pamper-project.com) and on social media (X: PAMPER_Project).

## Supplementary material

10.1136/bmjopen-2025-103763 online supplemental file 1

10.1136/bmjopen-2025-103763 online supplemental file 2

10.1136/bmjopen-2025-103763 online supplemental file 3
