# Supplementary material for: Development of a Physical Activity Maintenance intervention for people with PERsistent musculoskeletal pain (PAMPER): a mixed-methods study protocol
Source: BMJ Open. 2025 Jun 10;15(6):e103763. doi: 10.1136/bmjopen-2025-103763 (PMC12161382; doi:10.1136/bmjopen-2025-103763)
Supplement: online supplemental file 1 [file bmjopen-15-6-s001.docx]

**Topic guide PAMPER phase 1 (patient and physical activity partner)**

**Warm up questions and experience of physical activity since the pain management programme**

*First, I’ll ask some questions about your current physical activity and how you have found it since the pain management programme.*

Can you tell me what physical activity you do?

Where do you do your physical activity?

*Prompt: such as at home, leisure centre, a class?*

When we first spoke on the phone, you told me you were [consistently active, inconsistently active, inactive], can you tell me more about how much physical activity you do and when you do it?

How do you feel about keeping up physical activity long-term?

How have you found doing physical activity since finishing the pain management programme at [organisation]?

*Prompt: have you done as much as you wanted to? If yes, why? If not, why not?*

Is the physical activity that you do now different to when you were doing the pain management programme?

*Prompt: if so, what is different and why?*

**Barriers, facilitators, and intervention components**

*I am now going to ask you about what helps and hinders keeping up your physical activity.*

What makes keeping up your physical activity difficult? *(open question and discuss answers in more detail before going on to ask more specific questions below)*

*Prompt: is there anything else you can think of that makes keeping up physical activity difficult for you?*

What helps you to keep up your physical activity? *(open question and discuss answers in more detail before going on to ask more specific questions below)*

*Prompt: is there anything else you can think of that helps you to keep up your physical activity?*

*Planning, and intentions/routine/competing demands/burden*

*Next, I’ll ask you about your goals and plans for physical activity and how it fits into your life.*

Do you have a goal of how much physical activity you want to keep doing or what you want to gain from it?

*Prompts:*

- *If yes, why? What is your goal?*
- *If not, why not?*

How important are these goals for you?

How often do you achieve these goals?

*Prompts:*

- ***If achieves goals:*** *how confident are you that you can continue to meet your goal? What influences your confidence?*
- ***If does not achieve goals:*** *how confident are you that you can achieve these goals in the future? What influences your confidence?*
- ***If sometimes achieve goals:*** *how confident are you that can achieve these goals more often? What influences your confidence?*

Do the goals change?

*Prompts:*

- *If yes, why? What changes about them?*
- *If not, why not?*

Are you planning to keep up regular physical activity long-term?

*Prompt: Why do you plan (or not plan) to keep it up?*

Are your plans to do physical activity different now or the same compared to when you did the pain management programme?

How much do you plan your physical activity?

*Prompt: do you have a structure to when and how you do your physical activity? If so, does it help? If so, why? If not, why not?*

How does (or would) physical activity affect and fit into your lifestyle?

*Prompt: Are there any other roles or responsibilities that you have that affect your physical activity? For example, work or family. If so, how do these affect your physical activity?*

Do you ever have to decide between doing physical activity or something else?

*Prompt: For example, other pain management strategies, working or seeing family. If so, what do you tend to prioritise? How do you make these decisions?*

How do you find doing physical activity along with your other pain management strategies?

*Prompt: do you find it difficult to use multiple pain management strategies. If yes, why? If not, why not? How do you prioritise them?*

Can you think of anything that might help you or other people prioritise their physical activity or be able to keep it up despite other roles and responsibilities?

Have you ever identified as a physically active person?

*Follow-up: If yes, how important is this for your long-term physical activity? If not, do you think this impacts your physical activity? Has this changed since the pain management programme?*

*Beliefs about capabilities/confidence related to long-term PA*

*I’m now going to ask you about your confidence for keeping active long-term.*

How confident are you that you can keep up physical activity long-term?

How confident are you that you can get back to your desired level of physical activity if you have a dip in your activity or period of not being active?

What influences your confidence to keep up physical activity or get back to it if you have a dip?

How important do you think confidence is when keeping up physical activity? Why?

How confident are you that physical activity will be beneficial for you in the long-term?

*Prompt: If confident, why? If not, why not?*

How can people build their confidence to keep physically active, or keep confident to stay active?

*Feelings/experiences about maintaining PA*

*Now I’d like to ask you about your feelings around physical activity.*

How do you feel when doing physical activity?

*Prompt: do you feel anything good (like enjoyment) or bad (like fear).*

*Follow-up: Have these feelings changed since you first started doing physical activity with pain? If so, how?*

*Follow-up: Do these feelings influence how much physical activity you do?*

*Follow-up: How do you manage these feelings?*

*Problem solving, memory regarding PA and monitoring PA*

*I’m now going to ask you about times when it is difficult to keep up physical activity and about remembering to do it.*

In situations when it is more difficult to keep up your physical activity, how much of your physical activity are you able to do?

*Prompt: The same, less? Why?*

Are these situations the same or different to when you were increasing your physical activity during the pain management programme?

What do you do, if anything, to help you keep up your physical activity in these situations?

Can you think of anything that might help people to keep up their physical activity in these situations, or recover from periods where they are less active?

Do you ever have problems remembering to do your physical activity? If yes, why? If not, why not?

Is there anything you do to help you remember or that reminds you to do your physical activity?

*Prompt: for example, alarms on your phone.*

*Prompt: are these helpful? Do they work to remind you?*

*Prompt: can you think of anything else that could remind people to do their activity?*

How much has your physical activity become a habit?

If habitual:

- How much does this help you remember to do physical activity?
- Does this affect how much effort it takes to be active?
- Did you deliberately try and form the habit, or did it just happen?
- What did you do to try and make it a habit?

If not habitual:

- Do you think it would be helpful to be more of a habit?
- What do you think could make it more of a habit?

*Prompt: what do you think about adding it to an existing habit so that it becomes related to that habit? (e.g., taking a walk after a meal).*

Do you use anything to monitor or keep track of how much physical activity you do?

*Prompt: for example, a diary or fitness watch?*

*Follow-up: if so, does it help? If so, why? If not, why not?*

*Knowledge and beliefs about keeping up physical activity*

*I’ll now ask you about your knowledge and thoughts about keeping up physical activity.*

Do you know the benefits of keeping up physical activity?

*Prompt: Could you tell me what the most important ones are for you, that most motivate you to be active?*

*Prompt: do you think there are any downsides to keeping up physical activity? If so, what do you think they are?*

Do you think there are any benefits or downsides to not keeping up physical activity?

How important do you think consistency is when keeping up physical activity?

*Prompt: What do you think the impact of dips in physical activity level or periods of being inactive might be? Why?*

Do you know how to keep up physical activity?

*Prompt: do you know what to do, how much to do, where you can do it and how you can do it?*

What is important for people to know about keeping up physical activity with persistent musculoskeletal pain?

How could people with persistent pain be supported to improve and maintain their knowledge about physical activity?

*Social influences and communication*

*I’m now going to ask you about any support you receive for physical activity.*

Can you think of anything anyone has said or done that made you more motivated to be physically active?

Can you think of anything anyone has said or done that made you less motivated to be active?

**If individual interview:**

Do you have anybody that gives you support to keep up your physical activity?

*Prompt such as family, friends, healthcare professionals?*

*Prompt: if so, how do they support you? How important is this support?*

*Prompt: if not – how beneficial do you feel support from other people could be? Who do you feel that support would be beneficial from? And what could that look like?*

If at all, how do you talk to other people about your physical activity?

*Prompt: what do you talk to them about or tell them?*

How important is communicating with other people about keeping up your activity?

*If important, why? If not, why not?*

If they get support for PA: how did you go about getting this support for your physical activity? How could others get support for keeping up physical activity for themselves?

**If dyadic interview:**

In what ways does [name of PA partner] support you to keep up physical activity?

*Prompt if PA partner is someone they do PA with: What happens if [name of PA partner] is not available?*

How important is this support to you for keeping up your physical activity?

Do you have any other people who support you to keep up physical activity?

*Prompt: if so, in what ways do they support you?*

*Prompt: how important is this support?*

How did you go about getting this support for your physical activity? How could others get support for keeping up physical activity for themselves?

**Intervention characteristics**

You have already told me what might help people with certain aspects of keeping up physical activity, such as [list intervention characteristics so far]. As you know, we are going to design a support package to help people keep up physical activity after a pain management programme. I am now going to ask you some more specific questions about how people with your condition could be supported to keep up their physical activity.

Can you think of anything else that might help people, like yourself, to keep up physical activity after a pain management programme?

What could the support package include?

Who would be best to provide the support?

How could the support work?

*Prompts:*

- *Would you prefer to receive support face-to-face, via telephone or video call, digital media or a different way? Why?*
- *You mentioned earlier that X may be helpful, how could that look in a support package?*
- *Would you prefer to get support in a group or on your own? Why?*
- *How would the content of the support be delivered to you? For example, digitally, such as website or app, in a workbook?*

Where would be best for the support to work?

*Prompts:*

- *At home? How and why?*
- *Community centre? How and why?*
- *Healthcare centre? How and why?*

When do you think you would need the support package?

How much support do you think you would need to help you keep up physical activity? Would this vary or always be the same? Why?

If the support package could be personalised, how could that work? for example, could it be tailored to symptoms, certain barriers, or physical activity levels?

**Ending the interview**

*We are now at the end of the interview. Is there anything you’d like to add to anything we have discussed or anything new you’d like to mention?*

*Thank you for participating in the interview. I will now stop the recording.*
