# Supplementary material for: Development of a Physical Activity Maintenance intervention for people with PERsistent musculoskeletal pain (PAMPER): a mixed-methods study protocol
Source: BMJ Open. 2025 Jun 10;15(6):e103763. doi: 10.1136/bmjopen-2025-103763 (PMC12161382; doi:10.1136/bmjopen-2025-103763)
Supplement: online supplemental file 3 [file bmjopen-15-6-s003.docx]

**WP2 FOLLOW-UP QUESTIONNAIRE**

**Study Title**

Factors associated with physical activity maintenance and potential intervention components to support physical activity maintenance: a prospective longitudinal pilot study

**Please answer all the following questions**

**Section 1: information about you**

1. What is your full name?
2. What month and year were you born? (MM/YYYY)
3. Are you currently working or studying? (Please tick – can select more than one)

Working (paid employment)

Volunteering

Studying

Not working due to pain

Retired

Not working due to other reason

1. Do you have anybody that supports you with your physical activity or that you do physical activity with? (Please tick one)

Yes

No

1. Have you had support from the pain management programme team with physical activity since you finished the programme? (Please tick one)

Yes

No

1. Are you married or do you live with a partner? (please tick one)

Married or living with a partner

Not married or living with a partner

**Section 2: physical activity related questions**

1. How important is it to you to maintain your intended level of physical activity? (Please tick one)

1. Not at all important

2. Slightly important

3. Moderately important

4. Very important

5. Extremely important

1. How motivated are you to maintain your intended level of physical activity? (Please tick one)

1. Not at all motivated

2. Slightly motivated

3. Moderately motivated

4. Very motivated

5. Extremely motivated

1. Do you have physical activity related goals? (Please tick one)

1. Yes

2. No

1. To what extent do you agree or disagree with the following statements:
2. Physical activity makes me feel better physically (please tick one)

1. Not at all

2. Slightly so

3. Moderately so

4. Very much so

5. Extremely so

1. Physical activity makes me feel better mentally (please tick one)

1. Not at all

2. Slightly so

3. Moderately so

4. Very much so

5. Extremely so

1. Physical activity helps me manage my pain (please tick one)

1. Not at all

2. Slightly so

3. Moderately so

4. Very much so

5. Extremely so

1. Physical activity helps my general health (please tick one)

1. Not at all

2. Slightly so

3. Moderately so

4. Very much so

5. Extremely so

1. Physical activity might harm me (please tick one)

1. Not at all

2. Slightly so

3. Moderately so

4. Very much so

5. Extremely so

1. I should not do physical activities which (might) make my pain worse (please tick one)

1. Not at all

2. Slightly so

3. Moderately so

4. Very much so

5. Extremely so

1. I am worried about doing physical activity incorrectly (please tick one)

1. Not at all

2. Slightly so

3. Moderately so

4. Very much so

5. Extremely so

1. My fear of physical activity has reduced since the pain management programme (please tick one)

1. Not at all

2. Slightly so

3. Moderately so

4. Very much so

5. Extremely so

1. I am able to manage my fear of physical activity (please tick one)

1. Not at all

2. Slightly so

3. Moderately so

4. Very much so

5. Extremely so

1. How confident are you that you can maintain physical activity if:
2. The weather is bothering you (please tick one)

1. Not at all confident

2. Slightly confident

3. Moderately confident

4. Very confident

5. Extremely confident

1. You are bored by the physical activity (please tick one)

1. Not at all confident

2. Slightly confident

3. Moderately confident

4. Very confident

5. Extremely confident

1. You feel pain when doing physical activity (please tick one)

1. Not at all confident

2. Slightly confident

3. Moderately confident

4. Very confident

5. Extremely confident

1. You have to do physical activity alone (please tick one)

1. Not at all confident

2. Slightly confident

3. Moderately confident

4. Very confident

5. Extremely confident

1. You do not enjoy it (please tick one)

1. Not at all confident

2. Slightly confident

3. Moderately confident

4. Very confident

5. Extremely confident

1. You feel tired (please tick one)

1. Not at all confident

2. Slightly confident

3. Moderately confident

4. Very confident

5. Extremely confident

1. You feel stressed (please tick one)

1. Not at all confident

2. Slightly confident

3. Moderately confident

4. Very confident

5. Extremely confident

1. Your mood is low (please tick one)

1. Not at all confident

2. Slightly confident

3. Moderately confident

4. Very confident

5. Extremely confident

1. You feel fatigued (please tick one)

1. Not at all confident

2. Slightly confident

3. Moderately confident

4. Very confident

5. Extremely confident

1. You can be seen by others (please tick one)

1. Not at all confident

2. Slightly confident

3. Moderately confident

4. Very confident

5. Extremely confident

1. How confident are you that you can recover from periods of lower activity or inactivity? (Please tick one)

1. Not at all confident

2. Slightly confident

3. Moderately confident

4. Very confident

5. Extremely confident

1. How confident are you that you can maintain your physical activity without close guidance from a health professional, fitness instructor or other professional? (Please tick one)

1. Not at all confident

2. Slightly confident

3. Moderately confident

4. Very confident

5. Extremely confident

1. How difficult do you find it to remember to do physical activity? (Please tick one)

1. Not at all difficult

2. Slightly difficult

3. Moderately difficult

4. Very difficult

5. Extremely difficult

1. Is maintaining physical activity something you do automatically? (Please tick one)

1. Not at all

2. Slightly so

3. Moderately so

4. Very much so

5. Extremely so

1. To what extent do you plan your physical activity? (Please tick one)

1. Not at all

2. Slightly so

3. Moderately so

4. Very much so

5. Extremely so

1. How difficult is it for you to prioritise your physical activity due to:
2. Work (please tick one)

1. Not at all difficult

2. Slightly difficult

3. Moderately difficult

4. Very difficult

5. Extremely difficult

1. Caring for family (please tick one)

1. Not at all difficult

2. Slightly difficult

3. Moderately difficult

4. Very difficult

5. Extremely difficult

1. Social activities (please tick one)

1. Not at all difficult

2. Slightly difficult

3. Moderately difficult

4. Very difficult

5. Extremely difficult

1. Managing health conditions other than pain (please tick one)

1. Not at all difficult

2. Slightly difficult

3. Moderately difficult

4. Very difficult

5. Extremely difficult

1. Hobbies (please tick one)

1. Not at all difficult

2. Slightly difficult

3. Moderately difficult

4. Very difficult

5. Extremely difficult

1. How difficult do you find it to pace your physical activity?

1. Not at all difficult

2. Slightly difficult

3. Moderately difficult

4. Very difficult

5. Extremely difficult

1. How difficult do you find it managing your pain symptoms so you can maintain physical activity? (Please tick one)

1. Not at all difficult

2. Slightly difficult

3. Moderately difficult

4. Very difficult

5. Extremely difficult

1. How often did you have a pain flare-up in the last 6 months? (Please tick one)

1. Weekly

2. Fortnightly

3. Monthly

4. Every 2 months

5. Every 3 months

6. Less often than every 3 months

7. Did not have a flare-up

1. On average, how long did your pain flare-ups last in the 6 months? (Please tick one)

1. 1-2 days

2. 3-4 days

3. A week

4. 2 weeks

5. Longer than 2 weeks

1. How helpful do you find it to think about the level of physical activity you did before your pain started? (Please tick one)

1. Not at all

2. Slightly so

3. Moderately so

4. Very much so

5. Extremely so

1. Are you able to access appropriate places to do physical activity (i.e. are places near you suitable or are you easily able to get there)? (Please tick one)

1. Not at all

2. Slightly so

3. Moderately so

4. Very much so

5. Extremely so

1. Do you have access to the equipment you need to maintain your physical activity? (Please tick one)

1. Not at all

2. Slightly so

3. Moderately so

4. Very much so

5. Extremely so

1. Can you afford (financially) to keep up the physical activity you want to do?

1. Not at all

2. Slightly so

3. Moderately so

4. Very much so

5. Extremely so

1. Do the instructors at physical activity groups or classes you go to understand your condition? (Please tick one)

1. Not at all

2. Slightly so

3. Moderately so

4. Very much so

5. Extremely so

You do not go to physical activity groups or classes

1. How much does social media help you to keep up your physical activity? (Please tick one)

1. Not at all

2. Slightly so

3. Moderately so

4. Very much so

5. Extremely so

1. How often do you adapt your physical activity when needed (e.g., when your pain is higher)? (Please tick one)

1. Always

2. Often

3. Sometimes

4. Not often

5. Never

1. How much do you monitor your physical activity (e.g., writing what you do in a diary)? (Please tick one)

1. Always

2. Often

3. Sometimes

4. Not often

5. Never

**Section 3: support maintenance of physical activity**

1. Since you finished the pain management programme, has there been anything that has helped you keep up your physical activity? If so, what is it?
2. Since you finished the pain management programme, has there been anything that has made it difficult for you to keep up physical activity? Is so, what is it?
3. We are developing support to help people with persistent musculoskeletal pain to keep up their physical activity after a pain management programme. What do you think would be good to include in that support package?
4. What do you think would be the best ways to deliver such a support package (e.g., face-to-face, using an app)?

**End of questionnaire.**

**Thank you for taking the time to complete this questionnaire.**
