# Supplementary material for: Development of a Physical Activity Maintenance intervention for people with PERsistent musculoskeletal pain (PAMPER): a mixed-methods study protocol
Source: BMJ Open. 2025 Jun 10;15(6):e103763. doi: 10.1136/bmjopen-2025-103763 (PMC12161382; doi:10.1136/bmjopen-2025-103763)
Supplement: Supplementary file 4 [file bmjopen-2025-103763.reviewer_comments.pdf]

PEER REVIEW HISTORY

BMJ Open publishes all reviews undertaken for accepted manuscripts. Reviewers are asked to complete a checklist review form and are provided with free text boxes to elaborate on their assessment. These free text comments are reproduced below.

ARTICLE DETAILS

Title (Provisional)

Development of a Physical Activity Maintenance Intervention for people with PERsistent musculoskeletal pain (PAMPER): mixed-methods study protocol.

Authors

Booth, Gregory; Bearne, Lindsay; D'Lima, Danielle; Hudda, Mohammed; Ussher, Michael

---

This article was not externally reviewed at BMJ Open. Protocols that have been independently assessed prior to submission to BMJ Open are usually fast-tracked to publication on the grounds that further substantial changes will not be possible. This independent assessment will usually be external, independent review for both a substantial grant award from a non-commercial or government funder and ethics approval.
